# Computational approaches and challenges in the analysis of circRNA data

**DOI:** 10.1186/s12864-024-10420-0

**Published:** 2024-05-28

**Authors:** Barry Digby, Stephen Finn, Pilib Ó Broin

**Affiliations:** 1 School of Mathematical and Statistical Sciences, University of Galway, Galway, Ireland; 2 Discipline of Histopathology, School of Medicine, Trinity College Dublin and Cancer Molecular Diagnostic Laboratory, Dublin, Ireland

## Abstract

Circular RNAs (circRNA) are a class of non-coding RNA, forming a single-stranded covalently closed loop structure generated via back-splicing. Advancements in sequencing methods and technologies in conjunction with algorithmic developments of bioinformatics tools have enabled researchers to characterise the origin and function of circRNAs, with practical applications as a biomarker of diseases becoming increasingly relevant. Computational methods developed for circRNA analysis are predicated on detecting the chimeric back-splice junction of circRNAs whilst mitigating false-positive sequencing artefacts. In this review, we discuss in detail the computational strategies developed for circRNA identification, highlighting a selection of tool strengths, weaknesses and assumptions. In addition to circRNA identification tools, we describe methods for characterising the role of circRNAs within the competing endogenous RNA (ceRNA) network, their interactions with RNA-binding proteins, and publicly available databases for rich circRNA annotation.

## Introduction

Circular RNAs were introduced to the lexicon of RNA biology as early as 1976, originally detected in pathogenic plant viroids [1], murine respirovirus (Sendai virus) [2], hepatitis $\delta$ virus [3] and RNA viruses recovered from the cytoplasmic fraction of eukaryotic HeLa cells [4]. Following these reports of single-stranded covalently closed viral RNA structures, evidence of alternative forms of circRNAs derived from precursor mRNA (pre-mRNA) splicing events began to emerge [5–7]. During the 1990s and early 2000s, several studies showed that circRNA-producing genes are widespread in eukaryotic cells [8–18] however, due to their lack of translation potential, researchers believed circRNAs to be discarded by-products of splicing events. During the initial adoption of next-generation sequencing (NGS) technologies, circRNAs remained largely unstudied – with the poly-A selection protocols in RNA-Sequencing (RNA-Seq) technologies preferentially selecting messenger RNAs (mRNAs). Recent advancements in bioinformatics methods coupled with a widening range of protocols to interrogate the transcriptome have enabled the detection of circRNAs, with interest in the field rejuvenated when a landmark study by Salzman et al. (2012) identified RNA transcripts containing ‘scrambled exons’ characteristic of circRNAs in hyperdiploid acute lymphoblastic leukaemia diagnostic bone marrow samples [19]. Subsequent studies by Jeck et al. (2012) [20], Hansen et al. (2013), [21] and Memczak et al. (2013) [22] identified thousands of circRNAs in metazoans. Moreover, Hansen et al., (2013) [21] and Memczak et al. (2013) [22] demonstrated that CDR1as and circSry competitively bind micro RNA (miRNA) sequences via complimentary sites within their mature spliced sequence, suggesting a regulatory role within the competing endogenous RNA (ceRNA) network. These foundational works ushered in a plethora of novel research on circRNAs characterising their origin, biogenesis, structure and functions [23–26].

circRNAs exhibit stage and tissue-specific expression [27–29] and are enriched in exosomes [30]. Coupled with their high level of stability in contrast to other RNA molecules, circRNAs represent a promising biomarker for disease; circ-ITCH for example, acts as a tumor suppressor in lung cancer by inhibiting the Wnt/$\beta$-Catenin pathway [31], and circPVT1 acts as an oncogene in head and neck squamous cell carcinoma, displaying overexpression in tumor samples harbouring TP53 mutations [32]. In addition to applications as a biomarker, circRNAs can be constructed to target and sequester overexpressed oncogenic miRNAs. The synthetically generated circRNA *CM21D* was produced via t-RNA splicing and administered in experimental glioblastoma models, inhibiting miR-21-5p thus restoring the expression of tumor suppressor genes [33].

Given their diverse role within cells, it is imperative to accurately identify and annotate the functions of circRNAs using computational methods in conjunction with sequencing data. Multiple bioinformatics tools exist for identifying circRNAs in RNA-Seq datasets via the detection of chimeric reads representative of circRNA back-splice junctions (BSJ). Once a circRNA has been identified, researchers are often interested in quantifying its expression, predicting it’s interactions with other small RNAs and RNA binding proteins (RBPs), examining the ratio of circRNA expression compared to its cognate parent gene, and performing differential circRNA expression analyses. In this review, we discuss the current landscape of bioinformatics tools for circRNA analysis encompassing circRNA identification and annotation, circRNA quantification, circRNA functional prediction, and highlight some of the computational challenges involved. Whilst this review focuses on the computational analysis of circRNAs, we briefly detail the origin, biogenesis and structure of circRNAs as this information is necessary for understanding the algorithms employed by circRNA identification tools. For additional details on circRNA biogenesis, degradation, translation, cellular transport, downstream interactions and evolutionary conservation, we direct readers to a selection of recent reviews [34–41].

Finally, we also provide an overview of the currently available circRNA databases containing rich annotations for circRNAs derived from various tissue sources and cell lines using RNA-Seq datasets, predicted miRNA and RBP targets using cross-linking and immunoprecipitation sequencing (CLIP-Seq) and circRNAs associated with diseases.

## circRNA biogenesis and structure

Canonical linear mRNA splicing involves processing pre-mRNA to remove intronic sequences and the joining together of exon sequences to form a mature mRNA transcript. This process is mediated via the spliceosomal machinery composed of small nuclear ribonucleoproteins which recognise conserved 5’ splice sites, 3’ splice sites and a branch site within the intronic sequence. Spliceosomal machinery binds to the intron’s upstream 5’ splice donor site, pairing it with the downstream branch site forming a lariat loop structure. Following this, the downstream 3’ splice acceptor site of the intron splice site is brought in close proximity to the 3’ end of the exon, where, via a process of transesterification, the exons 3’ hydroxyl group attacks the phosphodiester bond of the 3’ intron splice acceptor site, covalently joining the exons, producing a mature mRNA and releasing the intron lariat structure [42].

**Fig. 1:**
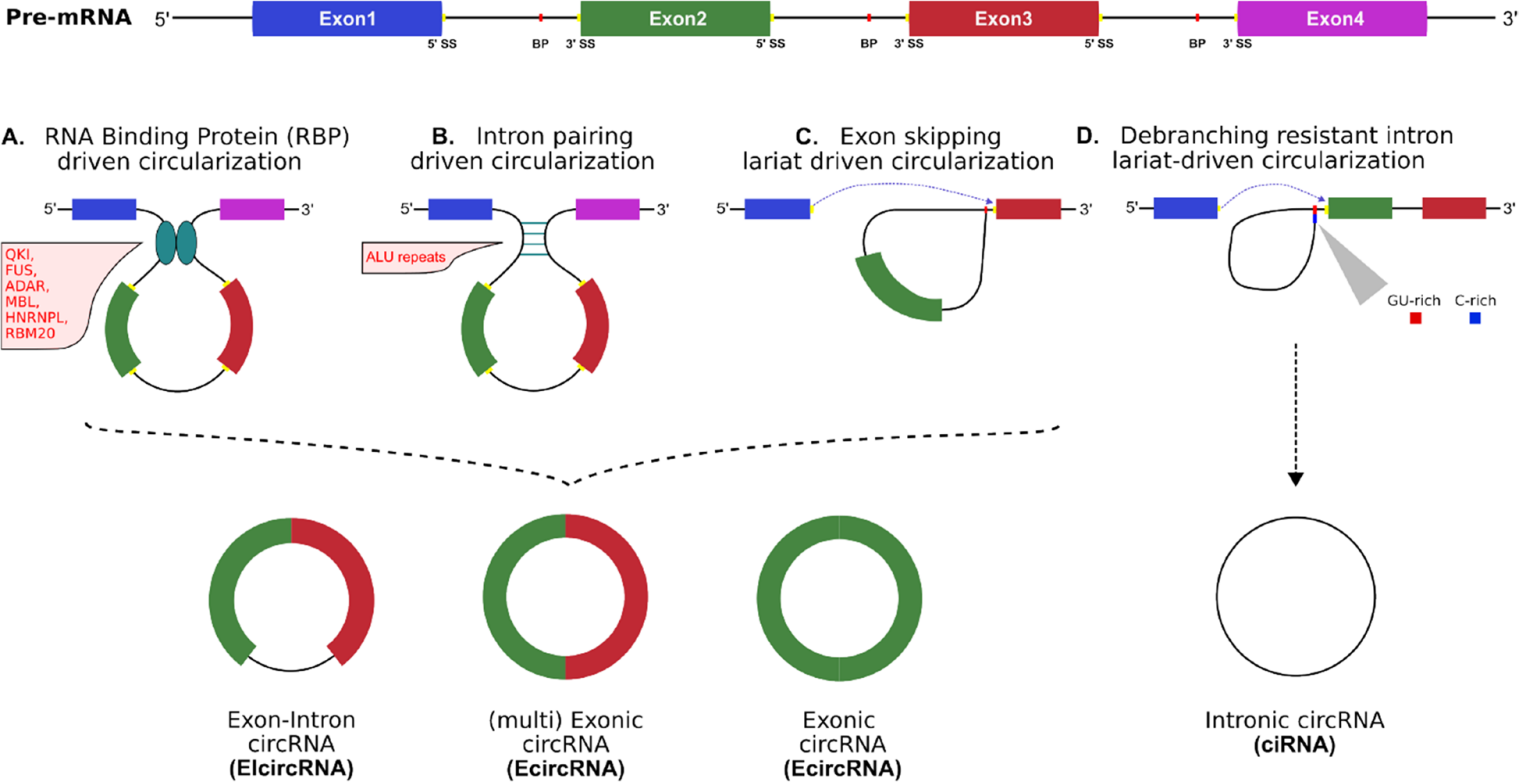
Biogenesis of circRNAs. **A**: RNA binding protein circularization, **B**: Reverse complementary matching sequence circularization e.g ALU repeats in humans, **C**: Lariat-driven circularization, **D**: Intron lariat circularization. Back-splicing processes **A, B** and **C** may undergo additional splicing to remove intronic or exonic sequences

circRNA formation relies on canonical splice site signals and spliceosome machinery [43] however, in contrast to linear RNAs, circRNAs are formed by a process known as back-splicing in which a downstream 5’ splice donor site is reversely joined to an upstream 3’ splice acceptor site forming a covalently closed loop structure [20, 24]. Similarly to linear RNAs, circRNA formation is regulated by *cis/trans* elements, and can be categorised as exonic circRNAs (EcircRNAs), exon-intron circRNAs (ElciRNAs) or circular intronic RNAs (ciRNAs) based on both the genomic position and the circularization process from which they were derived. Several RBPs act as *trans* factors inducing circRNA biogenesis including quaking (QKI) [44], muscleblind (MBL/MBNL1) [25], and fused-in-sarcoma (FUS) proteins [45]. By binding specific motifs in the intronic sequences flanking exons, dimerization of RBPs bring the downstream 5’ splice donor site in close proximity to an upstream 3’ splice acceptor site, facilitating back-splicing and the formation of exon-intron circRNAs (EIcircRNAs) or exonic circRNAs (EcircRNAs) (Fig. 1A). Reverse complimentary matches in flanking non-coding *cis* regulatory elements (e.g ALU repeats) can form a hairpin structure that bring the downstream 5’ splice acceptor site in close proximity to the upstream 3’ acceptor site facilitating back-splicing [20, 46, 47] (Fig. 1B). Circularization via intronic pairing is highly sensitive to both the composition and length of the hairpin structure — G-U wobbles and excessive stability hinders circRNA formation [48].

Lariat-driven circularization (exon skipping) is an event in which during canonical linear RNA splicing, the 3’ splice acceptor site of a distal exon is joined to the 5’ splice donor site of an upstream exon, forming a lariat loop structure containing skipped exon(s) (Fig. 1C). The lariat structure can then be processed to form an EcircRNA or EIcircRNA [13]. Notably, circulization of long exons (or a sequence of shorter exons) flanked by intronic regions of similar length hosting reverse complementary matches is favoured over shorter exons (average circRNA exon length 670nt [20] vs 120nt in mRNA [49]) [50, 51]. Finally, intron lariats that successfully circularize via the joining of a 11nt C-rich motif in the 5’ splice site donor sequence to the downstream 7nt GU-rich motif in the branch point site form a nascent ciRNA (Fig. 1D). The RNA structure formed by these specific motifs at the 5’ splice site and branch point site are resistant to debranching, releasing a stable ciRNA [52, 53].

circRNAs’ unique covalently closed loop structure lacking 5’ and 3’ tails confers resistance to RNase R degradation, granting them a much higher half-life than their linear mRNA counterparts [54, 55]. This feature makes circRNAs an attractive biomarker in disease-based settings, with reports of circRNAs exhibiting differential expression in gastric, colorectal, ovarian and lung cancers, and enzalutamide resistant LNCaP cell lines [56–58]. Furthermore, circRNAs can be packaged and exported from the cell to bodily fluid via exosomes [30, 59] facilitating the use of non-invasive liquid biopsies to monitor disease progression [60–65]. The mechanism of circRNA degradation and clearance remains an active area of research. Studies have found that miRNAs can facilitate the degradation of circRNAs via Argonaute 2 (Ago2)-mediated degradation supporting the hypothesis of circRNAs as active members in the ceRNA network [66]. Other works demonstrate RNase H1-mediated degradation of ciRNAs with high GC content [67] and the degradation of circRNAs containing m^6^A modifications via endoribonucleolytic cleavage [68].

## Principles and challenges for circRNA identification

### Library preparation

**Fig. 2:**
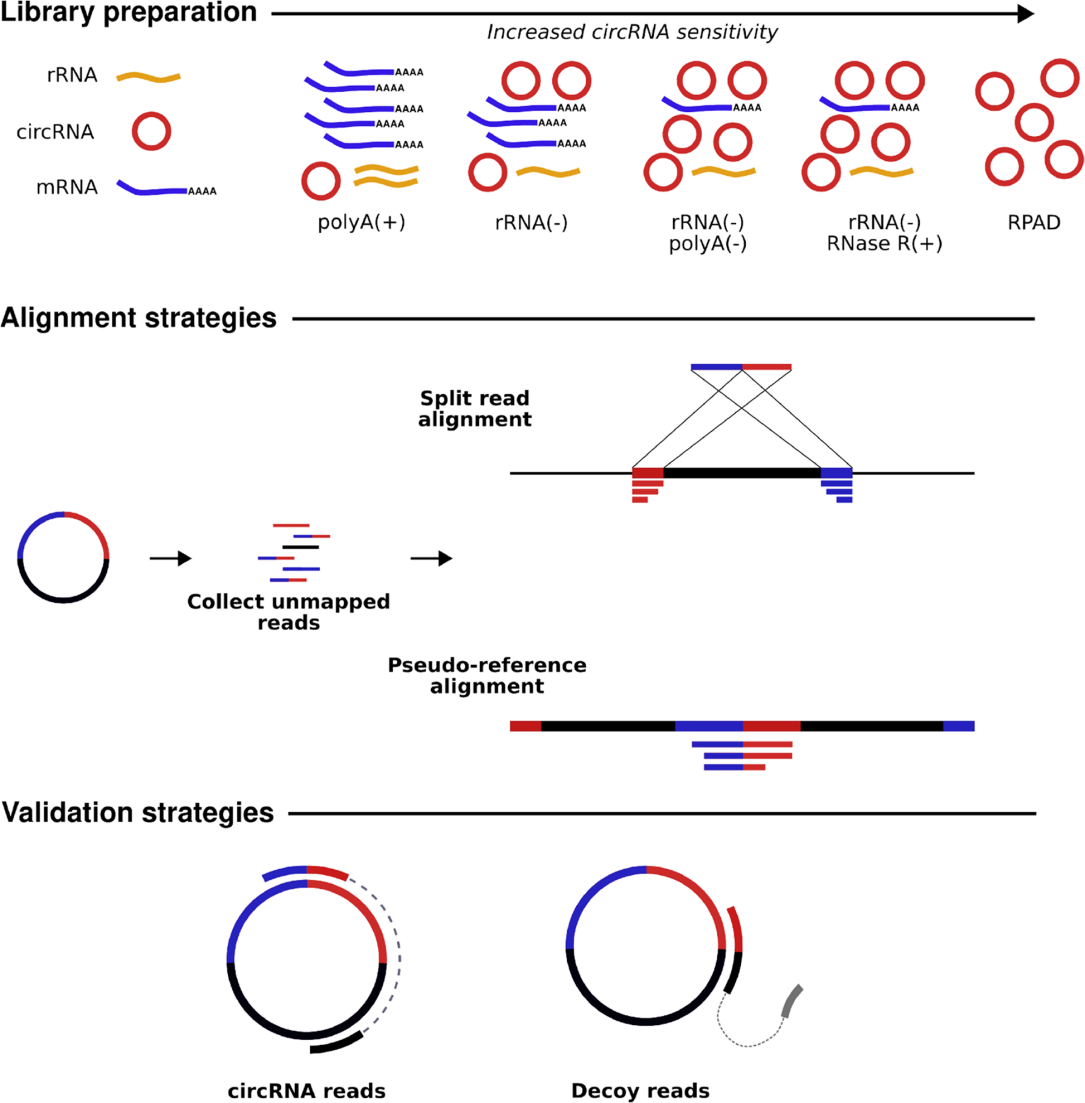
Advancements in biochemical and bioinformatics strategies for circRNA detection. Library preparation: Left to right, in order of increasing circRNA sensitivity; poly(A)(+): unsuitable for circRNA detection, preferentially selects mRNAs; rRNA(-): ribosomal RNA depletion yielding a library with circRNAs and mRNAs; rRNA(-) & poly(A)(-): ribosomal RNA depletion in conjunction with polyadenylation and depletion of poly(A) transcripts; rRNA(-) & RNase R(+): ribosomal RNA depletion in conjunction with RNase R ribonuclease treatment depleting mRNAs; RPAD: RNase R treatment followed by polyadenylation and poly(A)^R^NA depletion, yielding a highly pure circRNA library. Alignment strategies: Unmapped reads to the reference genome can be used to perform split alignment to identify reads aligning in opposite directions, or to map reads to a BSJ pseudo-reference. Validation strategies: Paired-end sequencing greatly reduces false-positives, mates that map across the BSJ whose mates reside within the same transcript are indictive of circRNAs

circRNAs represent approximately 1% of the transcriptome pool when compared to poly(A) RNA molecules [69], dictating novel strategies to enrich circRNA libraries prior to sequencing (Fig. 2). A typical eukaryotic RNA-Seq library preparation protocol involves the preferential selection of RNAs with poly(A) tails or the depletion of ribosomal RNAs (rRNAs). Due to circRNAs’ covalently closed loop structure, poly(A) selection in libraries will almost completely remove all circular transcripts in a sample. By contrast, circRNAs are retained in rRNA-depleted samples and are enriched in samples treated with ribonuclease R (RNase R) to deplete linear RNAs. Random priming is preferred to oligo(dT) priming, as the former generates random oligonucleotide sequences for cDNA synthesis independent of poly(A) sequences, whilst the latter will generate libraries biased towards linear RNAs. One method, termed "RNase R treatment followed by polyadenylation and poly(A)^+^ RNA depletion" (RPAD) has emerged as a leading candidate for circRNA library preparation yielding the highest number of circRNAs and the highest sensitivity in a benchmark study [70]. RPAD employs the sequential depletion of linear RNAs via RNase R treatment, polyadenylation of remaining linear RNAs and a final round of poly(A)^+^ depletion using oligo(dT) beads followed by ribosomal RNA (rRNA) depletion to yield a high concentration circRNA library for sequencing [71, 72]. In the absence of the RPAD method, rRNA depletion or RNase R^+^ are sufficient for generating RNA-Seq datasets for circRNA detection and have been used in benchmark studies analysing the performance of circRNA identification tools [73].

### Sequencing artefacts

Technical artefacts introduced during sequencing can lead to the generation of false positives during circRNA identification. Reverse transcriptase, an enzyme used to synthesise complementary cDNA strands can undergo a template-switching event when brought in close proximity to a different RNA template with a suitable region for priming [74]. The original, incomplete synthesized strand is carried to the newly ’switched‘ cDNA template where reverse transcriptase continues generating a chimeric molecule capable of mimicking alternative splicing and backsplicing. Alarmingly, these template switching events can account for up to 35-55% of the isoforms computationally detected for a gene [75]. Sequencing libraries that use adapter-ligation steps are at risk of generating chimeric sequences – albeit at a much lower level. Finally, incorrectly called bases at the beginning or end of exons in genes containing highly homologous sequences can generate false positive splice site signals (GU/AG, etc.) [76]. With respect to circRNAs, these sequencing errors can lead to sites that are mistakenly identified as backsplice sites when identifying circRNAs in samples. Due to the low levels of circRNA expression in cells when compared to other RNA transcripts, the presence of sequencing artefacts cannot be overcome by simply applying a read depth filter to quantification results. circRNA identification tools typically require paired-end data to overcome this source of error by requiring read 1 to map to the back-splice junction (BSJ) and its corresponding read 2 pair to map in the same transcript within a fixed distance [19].

### BSJ-based circRNA identification

The main step of any circRNA analysis is the identification of circRNAs in RNA-Seq datasets. This analysis is predicated on the detection of the BSJ, i.e the scrambled exon junctions representing the joining of an upstream 5’ donor site to the downstream 3’ acceptor site to form a circular structure. The majority of circRNA identification tools can be classed as one of two sub-groups: segmented-based-approach, whereby anchors (fixed-length segments taken from the end of reads) are extracted from unmapped sequencing reads and re-mapped to the genome; or pseudo-reference based in which a custom database of manually curated BSJ sites are generated and used to map the sequencing reads (Fig. 2). The first strategy allows for *de novo* circRNA identification whilst the second is constrained to exons contained within the reference annotation file used for constructing the pseudo-reference database. As circRNA identification tools evolved, developers began to blend the two approaches and incorporate BSJ sequence context to optimize the process of circRNA identification (Table 1).

**Table 1:** Bioinformatic tools for circRNA identification, quantification, isoform detection, full circle reconstruction, target prediction and differential expression analysis

Tool name	Description^a^	Installation^b^	Aligner^c^	Language	Ref
ACFS	Identification & quantification of circRNAs	Manual	BWA, BLAT	Perl	[77]
ACValidator	Assembly based circRNA detection	Manual, pip	BWA, Bowtie2	Python2	[78]
ANNOgesic	Archael/bacterial circRNA identification	Manual, pip3, Docker	Segemehl	Python3	[79]
AutoCirc	Fast identification of circRNAs	Manual	Bowtie2	C++,Perl	[80]
BIQ	Identify circRNAs using k-mers spanning BSJ	Manual	k-mer	C++,Perl, JavaScript	[81]
CircAST	Full circle reconstruction, assemble & quantify circular isoforms	Manual	TopHat	Python2	[82]
CircDBG	De Bruijn graph detection of circRNAs	Manual	k-mer	C++	[83]
circDeep	circRNA ifdentification using deep learning	Manual	k-mer	Python3	[84]
CIRCexplorer	Identify, quantify & annotate circRNAs	Conda, pip, BioContainers	STAR, TopHat	Python2	[47]
CIRCexplorer2	Identify, quantify & annotate circRNAs with updated *De novo* module	Conda, pip, BioContainers	BWA, MapSplice, Segemehl, STAR, TopHat	Python2	[85]
CIRCexplorer3	Compare circRNA & linear expression	Manual	Hisat2, StringTie, CIRCexplorer2	Python3	[86]
circFL-seq	Detect circRNA isoforms from nanopore reads	Manual, pip	minimap2	Python3	[87]
circLGB-circMRT	Predicting circRNA regulatory interactions by machine learning	Manual	*NA*	Python2	[88]
circMeta	Downstream functional analysis of circRNAs	devtools	*NA*	R	[89]
CircMarker	Fast identification of circRNAs	Manual	k-mer	C++, JavaScript	[90]
CirComPara	Automated detection of circRNAs usign integrated approach	Manual, Docker	Multiple	Python2, R	[91]
CirComPara2	Automated detection of circRNAs using integrated approach	Manual, Docker	Multiple	Python2, R	[92]
CircPro	circRNA coding potential using RNA-Seq & Ribo-Seq reads	Manual	BWA	Perl	[93]
circRNA_finder	Identification of circRNAs	Conda, BioContainers	STAR	Perl	[27]
circRNAFisher	Identification of circRNAs using statistical framework	Manual	Bowtie2	Perl	[94]
circRNAprofiler	Downstream functional analysis of circRNAs	BiocManager	*NA*	R	[95]
circRNAwrap	Automated detection, abundance, isoform & DE analysis	Manual	Multiple	UNIX	[96]
circtools	Suite of tools to identify, quantify, visualise & perform DEA	Docker, pip3	STAR	Python3,R	[97]
circseq_cup	Full-length characterisation of circRNAs	Manual	TopHat, STAR, Segemehl	Python2	[98]
CircSplice	Detect alternative splicing events	Manual	STAR	Perl	[99]
CIRI	Identification & quantification of circRNAs	Manual	BWA	Perl	[100]
CIRI2	Identification & quantification of circRNAs	Manual	BWA	Perl	[101]
CIRI-AS	Alternative circRNA splicing	Manual	BWA	Perl	[102]
CIRI-deep	Predict differentially spliced circRNAs	Conda	*NA*	Python3	[103]
CIRI-full	Full circle reconstruction	Manual	BWA	JavaScript	[104]
CIRI-long	Identify circRNAs in Nanopore reads	Manual, pip	BWA, minimap2	C++, Python3	[105]
CIRIquant	Identification, quantification, RNAase R correction, DEA of circRNAs	Manual, pip	BWA, HISAT2	Python2	[106]
CIRI-vis	Visualising circRNA alignments & isoforms	Manual	*NA*	JavaScript	*NA*
CirRBP	Stacked generalization ensemble deep learning model to identify RBP binding sites	Web server	*NA*	*NA*	[107]
CirRNAPL	circRNA identification using machine learning	Web server	*NA*	*NA*	[108]
CRIP	Predict circRNA-RBP interactions using neural networks & stacked codon-encoding	Manual	*NA*	Python3	[109]
CYCLeR	Reconstruct & quantify circRNAs	Docker	BWA, STAR, kallisto	R	[110]
DCC	Identify & quantify circRNAs	Conda, BioContainers	STAR	Python2	[73]
DEBKS	Downstream differential circRNA analysis	Conda, pip	*NA*	Python3	[111]
DeepCirCode	circRNA ifdentification using deep learning	Manual	*NA*	Pyton2, R	[112]
exceRpt	extracellular circRNA profiling	Manual, Docker	Bowtie2, STAR	JavaScript, UNIX	[113]
FcircSEC	Full sequence reconstruction	CRAN, devtools	*NA*	R	[114]
find_circ	Identification & quantification of circRNAs	Conda, BioContainers	Bowtie2	Python2	[22]
FUCHS	Alternative circRNA splicing	Manual	*NA*	Python2, R	[115]
hppRNA	Workflow for mRNA, lncRNA, circRNA identification & quantification	Manual	STAR	Perl	[116]
isoCirc	Full length circRNA isoform reconstruction	Manual, pip	Minimap2	[117]	
JEDI	circular RNA prediction based on junction encoders and deep interaction among splice sites	Manual	*NA*	Python3	[118]
KNIFE	Statistical based circRNA identification	Manual	Bowtie, Bowtie2	Python, R	[119]
MapSplice2	Splice-aware aligner	Conda, BioContainers	Bowtie	C++	[120]
miARma	circRNA quantification, miRNA targets & DEA	Manual	BWA	Perl	[121]
NCLcomparator	Screening of NCLscan results	Manual	*NA*	Python2, R, UNIX	[122]
NCLscan	Identification of non-co-linear transcripts	Manual	BWA, BLAT	Perl Python2	[123]
nf-core circrna	Autmated circRNA quantification, miRNA target prediction & DEA	Conda, Docker	Multiple	nextflow	[124]
Pcirc	Random forest plant circRNA identification	Manual	Bowtie2, TopHat2	Python3, R	[125]
PcircRNA_finder	Plant circRNA identification	Manual	*NA*	Perl, Python2	[126]
PRAPI	circRNA identification from PacBio Iso-Seq	Manual	GMAP	Python2	[127]
PredcircRNA	Classification of circRNAs using hybrid features	Manual	*NA*	Python2	[128]
PredcircRNATool	circRNA detection based on thermodynamic properties of flanking introns	Manual	*NA*	Python2	[129]
PTESfinder	Identify post-transcriptional exon shuffling events	Manual	Bowtie	JavaScript, UNIX	[130]
PFv2	Annotation free circRNA detection	Manual	STAR, Bowtie2	Python3, Java	[131]
RAISE	Identification, quantification & internal structure	Manual	Bowtie2, BWA, HISAT2, STAR, StringTie	UNIX	[132]
ROP	Identify RNAs in unmapped reads	Manual	*NA*	Python2	[133]
Segemehl	Short read mapper capable of detecting circRNAs	Conda, BioContainers	Segemehl	C++	[134]
StackCirRNAPred	Classification of circRNAs using Random Forest, LightGBM & XGBoost	Manual	*NA*	*NA*	[135]
STARChip	Identify circRNAs from STAR junction files	Manual	*NA*	Perl, UNIX	[136]
Ularcirc	Rshiny visualisation of circRNAs	devtools	*NA*	R	[137]
UROBORUS	circRNA identification	Manual	Bowtie	Perl	[138]

^a^*DEA* = Differential expression analysis

^b^*Manual*, requires one of source installation from GitHub, compilation using make, prerequisite software to be previosuly installed or a combination of all three. *BioContainers*, all Conda packages are automatically converted to container images hosted on BioContainers. Available via container clients such as singularity, docker etc

^c^*NA* refers to downstream tools that consume previously generated circRNA identififcation tool outputs as input, or classification tools that leverage experimentally validated interactions for prediction tasks

The first circRNA identification analysis performed by Salzman et al. (2012) [19] used the pseudo-reference based approach to identify circRNAs in ALL samples. Reads that mapped contiguously to RefSeq annotated genes using Bowtie [139] were considered representative of linear transcripts and removed from the analysis. Subsequently, the RefSeq database was used to create a custom database of all intragenic exon-exon junctions against which reads that failed to align were mapped. An exon scrambling event was flagged if read 1 mapped to a non-canonical exon-exon junction as defined by the custom RefSeq database and read 2 mapped within the same transcript. The number of reads spanning the scrambled exon junction was used to estimate the relative abundance of candidate circRNAs. In contrast to the pseudo-reference based approach, the first tool created for the purpose of circRNA detection ‘find_circ’ [22] utilises the segmented-based approach. Firstly, paired-end reads are aligned to the genome to extract reads that do not contiguously align. A customised script then splits unmapped reads to obtain 20 nucleotide anchor sequences originating from the 5‘ and 3‘ ends of the reads. The anchors are re-aligned to the genome, with anchors mapping in reverse orientation extended to identify the breakpoint site in the anchor. The resulting BED file is filtered using the following criteria to arrive at a set of circRNAs: 1) splice sites must be flanked by GU/AG signals; 2) unambivalant breakpoints; 3) less than 2 mismatches in the extension procedure; 4) breakpoint cannot reside more than 2 nucleotides inside the anchor; 5) more than 2 reads must support the BSJ site and 6) splice sites must not be more than 100Kb apart.

UROBORUS [138] adopts a similar approach to ‘find_circ’, collecting and extracting 20bp anchors from reads that failed to map contiguously to the reference genome using TopHat. Anchor segments representative of a circRNA BSJ site mapped in reverse orientation within the same transcript with an overhang of >20bp at either end of both segments are termed ‘balanced mapped junctions’ (BMJ) whilst segments with an overhang <20bp in one read are termed ‘unbalanced mapped junctions’ (UMJ) and subject to different extension strategies. Both BMJ reads are separately extended outwards to the nearest splice-site to form paired-end segments, whilst for UMJ reads, the shorter mate is discarded and the single mapped seed is outwardly extended to the nearest splice-site (an extension distance must not exceed the length of the read minus 3bp). Bowtie is then used to remap the paired-end and single extended segments; segments aligning to the reference genome in the opposite orientation with read support >2 as detected by the UROBORUS algorithm are representative of circular candidates.

Post-transcriptional exon shuffling finder (PTESfinder) [130] combines the segment-based approach with the pseudo-reference based approach to identify circular candidates using Bowtie. Briefly, 20bp segment anchors are extracted from the ends of input sequencing reads and mapped to the reference transcriptome. Anchors that map to the same gene but in an inverted orientation are identified and used to construct a pseudo-reference (termed ‘PTES constructs’) by concatenating the last 65bp of the underlying 5’ exon and the first 65bp of the 3’ exon. Reads are then aligned to the PTES construct, as well as genomic and transcriptomic references in order to generate mapping scores for circular candidate filtering. Candidates are marked as circRNAs when they exhibit high mapping scores to the PTES construct and low scores to the genomic and transcriptome reference.

The concept of mapping reads to genomic, transcriptomic and BSJ databases to filter circRNA candidates was further improved upon by KNIFE [119]. KNIFE maps reads to rRNA sequences, genomic, transcriptomic and a customised BSJ reference database using Bowtie2, discarding candidates that map with high scores to databases other than the custom BSJ reference. Where paired-end RNA-Seq data is available, the candidate reads spanning the BSJ site are subsetted into circRNA and decoy reads based on mapping information available in order to mitigate against false-positive BSJ reads generated by sequencing errors. For reads that fail to map to any of the databases, a *de novo* analysis is performed using Bowtie coupled with a segment-based approach whereby segments are used to construct a *de novo* index. The unmapped reads are then re-aligned to the *de novo* index using Bowtie2 with the same criteria for pseudo-reference based alignment. KNIFE is one of the first circRNA identification tools to employ a statistical framework by obtaining a posterior probability for each circRNA candidate to predict if it is a true positive by using a logistic generalized linear model (GLM) based on the alignment features of read 1. In contrast to the circRNA identification tools discussed thus far which require extracting anchor sequences to identify putative BSJ sites using Bowtie or Bowtie2, both BWA and STAR are capable of directly detecting breakpoint events and chimeric fusions during read alignment. circRNA identification tools utilizing BWA or STAR therefore circumvent the need to manually extract anchors for BSJ identification using customized scripts, streamlining the process of circRNA identification and reducing computational overheads.

CircRNA Identifier (CIRI) [100] is one such tool that utilises BWA-MEM sequence alignment mapping (SAM) information to identify reads in which two segments of the read align in chiastic order termed ‘paired chiastic clipping’ (PCC) signals. Subsequent filtering leveraging paired-end mapping (PEM) information, GU/AG splice signals and mapping rates to homologous sequences removes false positives to arrive at a set of high-confidence circRNAs. One shortcoming of CIRI is its handling of unbalanced junction reads. Unbalanced junction reads are segments of length <19bp which are ignored by BWA-MEM to prevent multi-mapping and erroneous mapping, therefore lacking the necessary alignment information in the SAM file for CIRI to detect PCC signals. CIRI uses a dynamic programming algorithm to re-map unbalanced junction reads to balanced junction reads originating from the same junctions detected in the first alignment step. This step is computationally expensive and leads to the generation of false positives, an area specifically addressed by its successor, CIRI2 [101]. CIRI2 is more cautious when addressing unbalanced junction reads and balanced junction reads with low mapping quality by utilising a maximum likelihood estimation (MLE) based on multiple seed matching. The undetermined segment of a putative BSJ read is divided into *n* seeds of length *m* (for example, a 50bp segment divided into five seeds of length 10bp) to determine if the segment belongs to a forward splice region or a back-splice region. The matched-seed numbers derived from the back-splice region (*k*_1_) and the forward-splice region (*k*_2_) are compared to produce two possible results *k*_1_ $k$_2_$=$ back-splice region; *k*_1_$\le$ $k$_2_$=$ forward-splice region. Since its publication in 2018, CIRI2 has become one of the most popular circRNA identification tools and has since been subsumed by CIRIquant [106] which extends its functionality by creating a pseudo-reference based on circular candidates detected by CIRI2, against which candidate reads are re-aligned using HISAT2. In addition to improved alignment, CIRIquant performs RNase R correction, linear RNA quantification and automated differential expression analysis of circRNAs. The suite of CIRI, CIRI2 and CIRIquant tools are all capable of calculating the ratio of circRNA BSJ reads and linear mRNA reads at a junction (CIRI2 and CIRIquant output this directly) to delineate the splicing preference in precursor mRNAs. When compared between conditions of interest, users can delineate differential splicing patterns.

Sailfish-cir [140] utilises the outputs of CIRI, KNIFE, circRNA_finder, or circRNA coordinates in BED format to transform candidate circular transcripts to pseudo-linear transcripts. Using the Sailfish model [141], Sailfish-cir estimates the expression of both linear and circular transcripts spanning the pseudo-reference. Users should be aware that Salifish-cir does not report BSJ counts, but rather outputs transcripts per million (TPM). Given both linear and circular TPM estimates are given for a junction, the junction ratio can be calculated manually for parent gene - circRNA ratio tests. ACFS [77] is another identification tool that uses BWA, however, its approach to circRNA identification is somewhat unorthodox. ACFS converts paired-end data to single-end data and collapses the reads prior to alignment, borrowing a strategy commonly used for miRNA alignment and quantification. After identifying candidate reads containing segments mapping in inverse orientation, ACFS uses maximum entropy models to predict the underlying BSJ sequence most likely to be generated by splicing. The advantage of this approach is that non-canonical dinucleotide splice sites are considered. The authors also point to the tool’s ability to detect fusion circRNAs generated by chromosomal translocation events. This raises the question as to how ACFS controls for sequencing artefacts which can mimic fusion events - particularly when the tool discards paired-end read information.

circRNA_finder [27] and CIRCexplorer [47] were the first tools to use the outputs from the STAR aligner to identify circRNAs. STAR is capable of directly detecting and writing chimeric reads to the output binary alignment map (BAM) file or separately to a junctions.out tab-separated text file when ‘–chimSegmentMin’ is set to a positive integer. Both circRNA_finder and CIRCexplorer take advantage of the lightweight junctions.out file which contains within each line the genomic coordinates and CIGAR flags corresponding to each read segment that comprise the chimeric RNA molecule. circRNA_finder imposes filtering on the putative circRNAs, allowing at most 3 mismatches, uniquely mapped reads, a maximum distance between splice-donor sites of 100kb and the condition that if one read spans the BSJ site, its mate should reside within the interval between the splice donor and acceptor site. Interestingly, CIRCexplorer does not impose such filtering strategies. It instead benefits from using an input reference gene annotation file to annotate putative circRNAs, thereby constraining results to exon-exon boundaries contained within the reference file, reducing the rate of false-positives. CIRCexplorer was superseded by CIRCexplorer2 [85], adding a suite of new modules for circRNA identification including alignment using TopHat-Fusion [142], annotation of circRNAs, *de novo* assembly of novel circRNAs, characterising alternative-splicing events within circRNAs and support for parsing BWA [143], MapSplice [120], STAR [144] and Segemehl [134] outputs. The deprecation of TopHat and TopHat-Fusion has resulted in CIRCexplorer2 largely becoming a tool for the downstream parsing and annotation of outputs from BWA, MapSplice, Segemehl and STAR. DCC [73] is yet another circRNA identification tool that harnesses the power of the STAR aligner. In its recommended workflow, paired-end mates are mapped using STAR and each individual mate is processed in the same manner, generating three output files per sample – joint mapping, mate1 and mate2 junctions.tab files. DCC also offers a junction ratio test using CircTest to formally test variation in expression between circRNAs and their parent gene. We have noted that the sensitivity of circRNA identification tools using STAR can be drastically increased by implementing STAR 2-Pass mode, in which the chimeric junctions detected in all samples during the first mapping stage can be collected and incorporated into the reference genome on the fly during the second pass mapping stage for a sample. This method comes at the cost of increased false positives [145] and as such we recommend users adopt an ensemble approach or set suitably strict filtering parameters on detected circRNAs when employing STAR 2-Pass mode with circRNA_finder, CIRCexplorer2 or DCC.

Finally, there exist splice-aware aligners that are capable of directly handling unmapped reads for detecting circRNAs during the alignment step. Non-co-linear scan (NCLscan) [123] and Segemehl [134] are two popular tools for this task, however, as NCLscan uses the proprietary aligner Novoalign, it’s use is dependent on an active Novocraft membership. For this reason Segemehl is considered the more popular tool in academic circles and has been incorporated into CIRCexplorer2 and intergration-based tools.

### Integration-based identification methods

A study by Hansen et al. (2016) [146] highlighted the discrepancies in results generated by the most popular circRNA identification tools at the time (circRNA_finder, CIRCexplorer, CIRI, find_circ and MapSplice). Strikingly, only 854 circRNAs were identified by all tools out of the 5071 unique circRNAs detected, indicating that the choice of circRNA identification tool drastically impacts analyses. Furthermore, the use of RNase R^+^ and RNase R^-^ libraries from the same samples permitted the calculation of false positives returned by each tool. By analysing each paired combination of circRNA identification tools, the authors show that circRNA_finder + CIRI had the highest rate of false positives (12.9%), whilst circRNA_finder + MapSplice achieved the lowest false-positive rate amongst analysed pairs (8.3%). Perhaps the biggest takeaway from the study was that the combination of all tools yielded a false positive rate of 6.56%, trading increased precision at the cost of reduced sensitivity. In 2018 Hansen [147] performed the same analysis again, this time using 11 circRNA identification tools (ACFS, CIRCexplorer, CIRCexplorer2, CIRI, CIRI2, DCC, find_circ, KNIFE, MapSplice and UROBORUS). Results echoed those from 2016, with Hansen providing the following key recommendation when adopting an ensemble approach: users should combine results from circRNA identification tools that utilise different aligners to avoid biases. One such example is circRNA_finder and DCC, which both use the STAR aligner. These two algorithms are thus less suited for pairing as the false positives generated are likely to be inherent to the aligner used. The analyses performed by Hansen et al. set new standards for best practices surrounding circRNA detection, ushering in a new class of circRNA identification pipelines termed ‘integrated tools’ in which the user can select one or multiple tools for circRNA identification analysis with an automated intersection of results based on user-defined parameters.

CirComPara, developed by Gaffo et al. (2017) [91] represents the first integration-based identification tool offering users the choice of CIRI, CIRCexplorer (STAR, BWA or Segemehl) and find_circ. Results are configurable by requiring detected circRNAs to have *n* reads spanning their BSJ site or circRNAs to be called by at least *n* tools. Requiring only input sequencing reads, a reference FASTA file and a reference annotation file, the workflow streamlines the process of circRNA identification for users by automatically generating the required genome indices, reformatting reference annotation files and executing scripts for the analysis. The authors have also made the considerable effort to create a docker container with all of the necessary software for the analysis included, circumventing the need to install any tools from source. Gaffo et al. made substantial upgrades to CirComPara in 2022 by releasing CirComPara2 [92]. In addition to offering updated circRNA identification tools to the user (CIRI2, CIRCexplorer2 (BWA, Segemehl, STAR, TopHat), DCC and find_circ), the workflow includes an improved expression estimate step when consolidating results from multiple tools. In CirComPara, circRNA abundances from multiple methods were calculated using the median of library-normalized BSJ counts across tools. In CirComPara2, the authors identify, for each method, the number of unique reads spanning the BSJ site of a circRNA thereby preserving the information returned by each tool used. Similar to CirComPara, CirComPara2 is packaged in a docker container facilitating rapid execution for users.

Several other integration tools exist for circRNA identification [89, 95, 96, 111, 114], however they operate by using as input previously generated results from circRNA identification tools, unlike CirComPara and CirComPara2 which produce results directly from raw sequencing reads. Another novel integration tool that works with raw sequencing data is nf-core circrna, a workflow for the quantification, miRNA target prediction and differential expression analysis of circRNAs [124]. The workflow takes as input raw sequencing reads, a reference FASTA, reference gene annotation file and performs all of the preprocessing steps and execution scripts required for a circRNA analysis using circRNA_finder, CIRIquant, CIRCexplorer2 (STAR), DCC, find_circ, MapSplice and Segemehl. Similarly to CirComPara, the user can specify custom filtering parameters dictating the intersection strategies used on results. With support for 18 species, the workflow additionally performs automatic miRNA target prediction using miRanda and TargetScan, and automated differential expression analysis of circRNAs between phenotypes of interest provided in an optional metadata file. Developed using nextflow DSL2, the workflow requires Java version >8, the latest version of nextflow and a container client which will automatically download software packages for each analysis step (Docker, Apptainer, Conda) facilitating rapid ‘out-of-the-box’ deployment using a single command.

### Full circle reconstruction

The first iteration of circRNA detection tools discussed above are predicated on identifying circRNAs via the presence of BSJ reads in sequencing data. Whilst this is an effective method to detect and quantify circRNAs in RNA-Seq data, the underlying mature spliced sequence (i.e the internal structure) of circRNAs remained opaque. circRNAs are subject to internal splicing events and intron retention (EIcircRNAs), therefore assuming that all of the underlying exons are retained within a circRNA will lead to false positives when predicting their targets based on sequence alignment against miRNA and RBP databases. To overcome this limitation and elucidate circRNA isoforms, coverage of paired-end RNA-Seq reads through the circRNA are utilised to characterise read densities amongst exons within the circular transcript.

The first tool developed capable of delineating circular isoforms via *de novo* construction of circRNA exons, CIRI-AS [102], was developed by the same group that produced CIRI2. Using the outputs from CIRI2 and a BAM file generated by BWA-MEM, the algorithm works by analysing local alignment positions of segments within candidate BSJ reads and its paired mate to identify forward spliced junctions representative of joined circular exons. For each circexon candidate, sequencing depth variation, BSJ read pair coverage and splice junctions from non-BSJ reads are taken into account. CIRI-AS can be performed without a reference GTF file, permitting flexible usage with non-reference organisms. In addition to detecting circexons, CIRI-AS can detect intronic or intergenic circRNA fragments (ICFs) when adequate sequencing depth is provided. CIRI-full [104] builds on CIRI2 and CIRI-AS for full resolution of circRNAs internal structure. The main premise of CIRI-full revolves around the detection of reverse overlap (RO) reads. During reverse transcription, the circular structure of circRNAs can cause continuous circumnavigation of reverse transcriptase within the circRNA, producing read pairs that overlap in reverse orientation. Moreover, the presence of a 3’-RO overlap in both RO reads indicates the full circle has been transcribed facilitating full circRNA reconstruction. For RO reads that do not overlap due to insert size length, CIRI-full borrows information from CIRI2 (BSJ sites) and CIRI-AS (circexons) to produce a reconstructed circRNA. Next, a forward-splice graph (FSG) is constructed by assembling BSJ and RO reads within a detected circRNA BSJ site to model the read coverage of each path using Monte Carlo simulations, providing resolution of circRNA isoforms.

Full characterization of circRNAs (FUCHs) [115] is yet another tool capable of detecting circular isoforms, accepting as input results from circRNA_finder, CIRI2, CIRCexplorer2, and DCC in conjunction with a BAM file containing chimeric reads, linear reads and unmapped reads. The first step is to isolate circular reads from the BAM file, then identify splicing events within the circular transcript by detecting exon-skipping events in reads. The coordinates of the skipped exons are used to generate coverage statistics, assigning reads to one of two circular isoforms. The output files generated detail the circular candidate’s genomic location coupled with read depth for each underlying exon. In this way, researchers can delineate the spliced transcript by removing exons with a read count of 0.

### circRNA identification using long-read sequencing

Long-read sequencing technologies developed by Oxford Nanopore Technologies (ONT) or Pacific Biosciences (PacBio) are capable of producing sequencing reads several thousand nt in length, providing full resolution of internal exon structure of linear transcripts [148–151]. This technology represents a promising avenue for full circle reconstruction of circRNAs over short-read based algorithms which struggle to identify circRNA FSJ sites located at distance from the BSJ reads [102, 104, 115]. However, in most cases cDNA sequencing is performed using oligo(dT) primers which are unsuitable for circRNAs lacking poly(A) tails, therefore requiring an adaptation of the amplification step prior to sequencing.

IsoCirc [117] is a strategy for characterising full-length circRNA isoforms using rolling circle amplification (RCA) followed by ONT sequencing. Here, samples are first treated with rRNA depletion and RNase R to deplete linear RNAs. Next, cDNA/circRNA double-stranded hybrids are generated using random hexamer priming in conjunction with reverse transcriptase, after which overhangs present in the cDNA circle are removed using Mung Bean endonuclease. The cDNA circle is then ligated using SplintR ligase to form a circular template for the generation of long concatemeric ssDNAs for sequencing via RCA. The strategy to generate concatemeric ssDNAs is a key step in the isoCirc protocol, as it allows for the generation of a final ‘consensus’ circRNA sequence thereby minimising the error rates associated with ONT sequencing [152]. Computationally, the consensus circRNA sequence is generated using Tandem Repeats Finder [153]. Two copies of all consensus sequences that pass filtering are concatenated and used for downstream mapping to the reference genome via minimap2 [154]. Subsequent filtering strategies are used to identify both the optimal alignment record per consensus sequence and the optimal consensus sequence per long read. Only candidate circRNAs with high quality BSJ and FSJ sites are reported as full-length circRNA isoforms, whilst single-exon circRNAs require only high confidence BSJ sites.

In contrast to the RCA amplification method employed by isoCirc, CIRI-long [105] utilises rolling circle reverse transcription (RCRT) to synthesise circRNA cDNA. First, circRNAs are enriched using a customised approach for RNA-seq library preparation. rRNA depletion is performed using a RiboZero kit followed by poly(A)-tailing prior to RNase R digestion to increase linear RNA degradation [155]. The remaining circRNAs in the library are amplified using random primers and SMARTer reverse transcriptase to initiate RCRT and cDNA synthesis. This step generates long cDNA fragments, within which exists multiple copies of full-length circRNA sequences. SMARTer sequencing adapters are added to each cDNA fragment to enable effective amplification in the absence of poly(A) tails. Once cleaned circRNA reads have been obtained, CIRI-long has two main steps: 1) candidate circRNA identification and 2) isoform colapsing. Step 1 involves using *k-mers* to search for repetitive sequences and the boundaries of circRNA fragments within the long reads. Next, a cyclic consensus sequence (CSS) for each read is generated using the SPOA library [156], with an 80% similarity score as defined by the Levenshtein distance used to filter high-confidence circRNA candidates. CSSs of length >150bp are then mapped to the reference genome using minimap2, whilst shorter reads are mapped using BWA MEM. An iterative alignment strategy is used during CSS alignment, whereby unmapped segments residing in the head or tail region of the CSS are appended to the opposite end of the CSS. Subsequent realignment determines if the re-ordered CSS obtains better scores than the previous alignment. This iterative process converges once the transformed CSS is fully aligned to the reference genome with high scores. In step 2, candidate circRNA isoforms are detected by clustering reads based on their location within the reference genome. All sequences within a cluster are subject to hierarchical clustering based on pairwise sequence similarity, producing consensus sequences for each cluster representative of a circRNA isoform.

circFL-seq [87] is another tool for detecting circRNAs using long reads, sharing similarities with CIRI-long in terms of library preparation and the generation of circRNA cDNA templates using RCRT. The bioinformatics component is divergent, relying on a pseudo-reference based approach after identifying consensus sequences. Reads are initially aligned to the reference genome using minimap2 to identify chiastic overlapping segments indicative of candidate circRNA reads (CCR). During this step, CCRs are classified as normal, fusion on same chromosome or fusion on different chromosome. The boundary of the chiastic segment of the CCR are used as a proxy for BSJs, and subsequently used to concatenate two sequences 150bp upstream to 150bp downstream of the BSJ to generate a pseudo reference sequence for each read. CCRs are then aligned against the pseudo reference, corrected using multiple aligned BSJ sites, reference annotations and canonical splicing motifs. Full length circRNAs are produced leveraging the BSJ and FSJ information for a given circRNA. circFL-seq offers two optional modules for low quality reads; *De novo* self-correction (DNSC): consensus sequences of reads are detected using TideHunter [157]. Following removal of consensus sequences containing long repetitive elements using Tandem Repeats Finder [153], a set of filtered consensus sequences are available for downstream processing. cRG mode: using the self-corrected CSs, RG mode is re-run using a query sequence of three copies of the corrected CS. The authors of circFL-seq found that cRG correction reduced the error rate of both indels and mismatches in the consensus sequence, and thus should be run for all deployments of circFL-seq.

In comparisons between the tools [87, 158], the RCA method was shown to produce longer reads than the RCRT method (up to 50kb vs. 1kb). Whilst more expensive, the longer reads produced by the RCA method allow for error correction during the consensus sequence identification step. Of note, the ligation step by isoCirc may introduce false positives via the ligation of residual linear RNA or truncated circRNA cDNA fragments that are difficult to resolve computationally. The RCRT method coupled with anchor priming or template switching employed by circFL-Seq and CIRI-long, respectively, are more resistant to this issue. In a direct comparison using HEK293 cells and mouse brain tissue, circFL-Seq was shown to be more sensitive than CIRI-long (HEK293: 27869 vs. 15242; mouse: 18396 vs. 9258 known BSJ sites), with similar rates of precision [87]. Conversely, in comparison with isoCirc with deep sequencing libraries, circFL-Seq was less sensitive than isoCirc (34046 vs. 79312 known BSJ sites). IsoCirc recovered far more circRNAs expressed at low levels (38846 vs. 2511, read count=1) indicating that whilst more expensive, isoCirc is the most sensitive method for detecting circRNAs from long-read sequencing.

### Machine learning circRNA identification

circRNA biogenesis can be attributed to hallmarks within the flanking intronic regions: reverse complimentary matching (RCM) sequences [159] (also referred to as inverted repeats [160]), and more specifically, ALU and tandem repeat motifs in humans [20] facilitating the generation of RNA hairpin structures that bring distal splice sites within close spatial proximity. These hallmarks coupled with evolutionary conservation scores, secondary structure information and the density of single nucleotide polymorphisms (SNP) within conserved miRNA binding sites [161] have been identified as predictive features for discriminating circRNAs from other classes of long non-coding RNAs (lncRNAs) using statistical and machine learning (ML) based approaches [84, 108, 128, 135]. Released in 2015, PredcircRNA [128] represents the earliest attempt at leveraging multiple layers of contextual sequence information to discriminate circRNAs vs. lncRNAs. The 188 features extracted from transcripts for training and testing the PredcircRNA model fall under one of four categories: 1) Graph features from RNA structures: nodes represent nucleotides and edges provide higher level information such as sequence backbone connection or bonds between base pairs [162]. To reduce the dimensions of the graph Random Forest (RF) was applied to extract the top 101 features. 2) Sequence conservation scores were computed using PhyloP conservation tracks [163], wherein the mean, variance, and maximum conservation scores within each transcript were determined. Additionally, the authors calculated the cumulative successive conservation score and the frequencies of nucleotides surpassing binned score thresholds. 3) Component composition scores: tri-nucleotide composition, GC content, sequence length, the presence of GT, AG, GTAG and AGGT motifs were extracted. 4) ALU, tandem repeats, ORFS and SNP: genome tracks for ALU sites, ORF sequences and SNP sites were downloaded and qunatified at the transcript level. The 188 extracted features were ranked in terms of importance using RF, with conservation features, GT/AG motifs and component composition scores identified as the most influential features for circRNA classification. The authors next utilised three machine learning classifiers, RF, support vector machines (SVM) and multiple kernel learning (MKL) to predict circRNAs, with the MKL method providing the best results in both the training and the independent test sets. Similarly to PredcircRNA, circDeep [84] leverages sequence features to classify circRNAs. The authors developed three novel descriptors to classify circRNAs; 1) RCM descriptor: a *H*-score which represents the presence of RCMs, 2) Conservation descriptor: utilising phastCons [164], the maximum, mean and median value of averaged conservation scores per exon are calculated (intronic transcripts are treated as a single exon) in addition to analysing the number of successive bases whose scores are above a given threshold, and 3) Asymmetric convolutional neural network - bidirectional long short-term memory network (ACNN-BLSTM) descriptor: a deep learning model that learns the local sequence context of transcripts as well as long-range dependencies between sequence features learned by ACNN layers. Using each of these three descriptors, the authors developed a fusion model to combine the three heterogeneous modalities termed ‘feature fusion fine-tuned’ which boasts greatly improved run times over PredcircRNA (largely due to the absence of GraphProt in the pipeline) and an impressive 12% increase in accuracy.

A limitation of these methods is that splice site and back-splice junctions are ignored, focusing instead on surrounding sequence context and classification tasks delineating mRNAs vs. lncRNAs vs. circRNAs. Given the unique BSJ of circRNAs, it is key to understand the properties and relationships between splice sites that constitute canonical linear splicing and a circular back-splicing event. DeepCirCode [112] analyzes the nucleotide sequences of two splice sites and predicts whether the two splice sites produce a back-splicing event characteristic of circRNAs. Briefly, the DeepCirCode model was trained using 50nt sequences surrounding each back-splicing instance in a custom dataset (back-splice sites detected by at least two computational methods present in circRNADb [165] or circBase [166]) and fed to a convolutional neural network (CNN). By leveraging the position weight matrices (PWMs) learned by DeepCirCode, users can predict the likelihood of a given sequence to produce a back-splicing event. Junction encoders and deep interation (JEDI) among splice sites [118] is a tool that optimizes a deep learning model for circRNA prediction in the absence of annotated back-splice sites as training data (zero-shot learning). Unlike its predecessors, JEDI is not limited to interrogating only two splice sites. In this way, it can model the sequence context and flanking regions of all splice sites within an transcript, making it an effective tool for classifying circRNAs that are derived from genes which also produce linear transcripts.

The latest addition to the suite of circRNA machine learning tools, CIRI-deep [103], infers differentially spliced circRNA (DSC) events between tissues/samples of interest in various types of datasets by leveraging the previously published Deep-learning Augmented RNA-seq analysis of Transcript Splicing (DARTS) framework [167]. Briefly, the CIRI-deep neural network model was constructed by running CIRIquant [106] on 397 filtered samples from RNA Atlas [168] and CircAtlas [169] to obtain back-splice junction (BSJ) counts and forward-splice junction (FSJ) counts representative of cirRNA and linear mRNA reads spanning junction sites. Dataset labels were generated in a pairwise fashion using DARTS Bayesian Hypothesis Testing with an uninformative prior (DARTS BHT-flat) wherein the junction ratios were used to assign high-confidence differential or unchanged splicing events between samples. Next, 3527 relevant circRNA *cis* sequence features were extracted in addition to the expression levels of 1499 *trans* RBPs associated with circRNA biogenesis-related genes, splicing factors and RNA degradation enzymes. The design and underlying model employed by CIRI-deep offers a two-fold advantage for users: 1) By developing a model trained on approximately 25 million DSC events and both *cis* and *trans* factors, CIRI-deep can predict DSC events independent of BSJ reads by incorporating a Bayesian prior (DARTS BHT-info). This permits the usage of CIRI-deep on datasets with low replicates, low sequencing depth and 10X single cell or spatial transcriptomics datasets in which circRNA BSJ reads are sparsely detected. 2) CIRI-deepA, a variant of CIRI-deep, was trained on *trans* RBP gene expression data from poly(A) selected datasets in RNA Atlas, permitting the detection of DSC events in large cohort datasets such as GTEx [170] and TCGA [171]. The authors of CIRI-deep conceed that the model is not without limitations, particularly in the context of cancer samples in which mutations to *cis* elements and dysregulation of *trans* factors are not suitable for use by a model trained on reference genomes.

### Overview of computational challenges

Hypotheses generated about the genome-wide role of circRNAs must be based on accurate quantification of circRNAs to mitigate the propagation of false positives in published literature. In this section, we discuss current computational methods used by researchers to arrive at a set of high confidence circRNAs.

#### circRNA detection strategies

Researchers should be aware that tools will generally fall under one of two categories: 1) segmented based circRNA detection or 2) pseudo-reference based circRNA detection. In the segmented based approach, unmapped reads (i.e reads that do not contiguously align to the reference) are collected and split into segments in order to identify reads that map to the back-splice junction. Whilst this method permits the discovery of *de novo* circRNAs and is more suited for organisms with incomplete or poorly annotated reference genomes, the method is less sensitive [24]. Researchers should therefore investigate the methods used by the quantification tool to mitigate these erroneous circRNA calls and if absent, apply filters manually. Possible methods for reducing false positives in segmented-based circRNA detection are: 1) Requiring the mate of a candidate BSJ read to be within a suitable distance and mapped within the same transcript. This method removes decoy reads generated by genomic rearrangements or sequencing artefacts that mimick the BSJ of circRNAs [27]. 2) Filter BSJ sites to keep candidates that are flanked by canonical splice site motifs (e.g GU/AG) [22]. Alternatively, users can inlcude non-canonical splice sites in their search and apply a ranking system e.g GC/AG U2-type, AT/AC U12-type [105] in order to score *de novo* circRNAs. 3) Enforce high quality mapping around the BSJ site, e.g requiring no more than *n* mismatches, insertions or deletions in *n*-bp each side of the BSJ junction. By combining metrics 2 and 3, researchers can produce a ranked list of circRNAs instead of applying hard filters.

For the pseudo-reference based approach, there are two methods by which a pseudo-reference database can be designed which will greatly influence the circRNAs detected by such a tool. The first method is to generate a database of all intragenic exon-exon junctions using the reference GTF/GFF file, thereby creating every possible combonation of back-splicing events. This method is restricted to species that provide an annotated reference genome file and can only detect circRNAs that share splice sites with linear RNAs. The second method is more favourable to unannotated organisms, whereby circular candidates collected in the first alignment step are tandemly duplicated to construct a pseudo-reference circRNA transcriptome against which the circular candidate reads are mapped against. This method reduces the rate of false positives by requiring the circular candidate reads to be linearly and fully aligned to the BSJ region of the pseudo-reference [106, 130, 140].

Due to the discrepancies in the two approaches, high variance between sets of circRNAs called by individual tools inevitably develops due to computational ‘blind spots’ inherent in each approach [172]. Users will therefore be tempted to apply multiple circRNA quantification tools to their chosen dataset, particularly with the advent of integrated based tools [91, 92, 96, 124, 132]. Whilst combinatorial approaches to circRNA identification will greatly increase the precision of the results, users should be aware that the sensitivity of different tool combinations will vary greatly [146, 147, 172–174].

#### circRNA identification tools exhibit high variance

Perhaps the most striking statistic that researchers will encounter when employing one or more circRNA quantification tools is the disparity in agreement amongst tools. Whilst individual tools have been shown to exhibit high precision, their ability to detect all true positive circRNAs in the benchmarking pool (i.e sensitivity) fluctuates [173, 174]. We describe three publications: Zeng et al. (2017) benchmarking 11 circRNA identification tools individually [173]; Gaffo et al. (2022) benchmarking combinations of 7 circRNA identification methods [92] and Vromman et al. (2023) benchmarking 16 circRNA identification tools in conjunction with orthogonal validation techniques [174] to highlight this point.

##### Zeng et al. 2017

The authors evaluated CIRCexplorer [47], circRNA_finder [27], CIRI [100], DCC [73], find_circ [22], KNIFE [119], MapSplice [120], NCLScan [123], PTESFinder [130], Segemehl [134] and UROBORUS [138] circRNA identification tools in order to assess the precision, sensitivity, F1 scores and AUC of each tool in both simulated and real datasets. In a simulated positive dataset containing 14689 HeLa circRNAs deposited in circBase [166], most tools achieved high precision (>94%). However, the sensitivity of each tool varied, ranging from 52%-93%. The authors then calculated the harmonic mean of precision and sensitivity (F1 score) to determine the best performing tools. Only four tools had an F1 score $\ge$0.9 — KNIFE (0.96), CIRI (0.92), PTESFinder (0.91) and Segemehl (0.91). KNIFE was the best performing tool in the simulated positive dataset, capturing 92% of the available circRNAs at a precision rate of 99.66%.

The authors then generated a background simulated linear RNA dataset to assess the fraction of false positive circRNAs called by each tool (NCLScan was omitted due to its inability to construct a noncollinear reference from linear reads). Alarmingly, Segemehl (1084), find_circ (712), UROBORUS (620) and KNIFE (554) called a high rate of false positive circRNAs in the background dataset. The simulated positive dataset was then added to the background dataset to generate a mixed dataset. Interestingly, Segemehl (87%), UROBORUS (88%) and find_circ (92%) exhibited dramatic drops in precision compared to the positive dataset analysis, whilst all other tools achieved precision rates above 96% in the mixed dataset. NCLScan boasted impressive precision rates in each dataset (99%) however this score is undermined by the fact NCLScan detected only $\sim$7740 circRNAs from the pool of 14689, reflected in poor sensitivity scores (52% positive and mixed). Using the simulated datasets the authors have demonstrated the underlying variance in sets of circRNAs called by each tool, underpinning the fact each tool has its own blind spots.

Next, the authors obtained HeLa RNaseR^-^, HeLa RNaseR^+^ and Hs68 RNaseR^-^, Hs68 RNaseR^+^ datasets with the goal of identifying the percentage of ‘true circles’ detected by tools in RNase R^-^ samples i.e called circRNAs that were not then depleted in RNase R^+^ samples. MapSplice, which had shown high precision and relatively poor sensitivity in the simulated datasets captured the highest percentage of true circRNAs (54% HeLa, 76% Hs68) indicating that whilst conservative, MapSplice captures a high proportion of true positives. Finally, the authors identified the top 100 expressed circRNAs identfied by each tool in the RNase R^-^ datasets to ascertain if high BSJ read counts are a reliable proxy for ‘true circles’ as performed by Hansen et al. 2016 [146]. In the HeLa dataset, KNIFE (75), CIRCexplorer (73), CIRI (72), circRNA_finder (72) and DCC (71) captured the highest proportion of true circRNAs exhibiting high expression. Conversely in the Hs68 dataset which had much higher coverage, eight of the circRNA identification tools top 100 circRNAs were well represented (>70) in the RNase R^+^ samples. This demonstrates high BSJ read counts are not necessarily indicative of true circRNAs, thus the common practice of applying BSJ count filters will not fully remove false positives. Furthermore, researchers should take caution when selecting circRNA identification tools for analysing sequencing libraries with lower coverage.

##### Gaffo et al. 2022

The demonstration of high variance in individual sets of circRNAs called by circRNA identification tools [146, 173] led the research community to experiment with combinatorial approaches in an effort to increase sensitivity without sacrificing precision. In 2018, Hansen et al. [147] performed a combinatorial analysis of 11 circRNA identification tools, concluding that combining tool outputs generally reduced the number of highly and lowly expressed algorithm specific false positive circRNAs. In 2022, Gaffo et al. [92] released CirComPara2, a tool that integrates seven circRNA identification methods (CIRI2 [101], CIRCexplorer2 [85] (parsing the outputs of BWA [143], Segemehl [134], STAR [144] and TopHat2/TopHat-Fusion [142, 175]), DCC [73] and find_circ [22] — circRNA_finder [27] and Segemehl [134] were omitted from the combinatorial analysis) to automate the identification of circRNAs from raw sequencing reads. To advise users on the optimal parameters required for running CirComPara2, the authors performed an analysis using both simulated and real datasets.

Firstly, a simulated dataset containing 5680 circRNAs was generated to characterise the false negatives in each identification method. On average 49% of the false negatives exhibited expression levels higher than the median expression level of true positives, whilst the expression level of false positives was low. Next, the authors identified the 1945 circRNAs that went undetected by one or more methods i.e the ‘false negative set’. They found that only 4% of the false negative set went undetected by all methods, whilst 95% of the false negative set could be detected using combinations of two or more methods. The results of the simulated analysis suggest that the computational ‘blind-spots’ (i.e inability to detect the false negative set) inherent in each method can be mitigated by supplementing results in concert.

To identify the optimal combination of methods for users to employ, the authors evaluated the number of recovered false negatives against the fraction of false positives introduced using all possible combinations of methods via precision, sensitivity and F1 score. Due to findings in the simulated dataset that combining circRNA_finder or Segemehl with other methods increased the number of false positives, these methods were omitted from the analysis. Unsurprisingly, the set of results produced by all individual methods yielded the highest sensitivity (0.99) and the lowest precision (0.90). Evaluation of sets produced by at least two conjoined methods yielded a marked increase in precision (0.99) at the cost of marginally reduced sensitivity (0.98). The authors demonstrate that increasing the required number of methods a circRNA must be called by (three or more conjoined methods) increases precision, however sensitivity drops considerably (0.96-0.69). The authors therefore recommend using all seven circRNA identification methods, requiring circRNAs to be called by at least two methods. This is the default setting for CirComPara2.

In our previous work [124], we observed a similar inflection point in F1 scores when combining the results of multiple tools (CIRIquant, CIRCexplorer2_STAR, circRNA_finder, DCC, find_circ, MapSplice, Segemehl). Our analysis revealed optimal F1 scores when using three or four quantification tools in addition to requiring circRNAs to be called by at least two methods. Increases in F1 scores were observed when using five or more tools, however the increased computational cost may not justify the marginal gains in precision. It should be noted that nf-core circrna users are discouraged from including DCC due to the high rate of false positives based on our findings.

Finally, the authors collected RNase R^-^ and RNase R^+^ datasets in human, mice and macaque species. Similarly to Zeng et al. 2017, true positives are defined as circRNAs detected in both untreated and treated matched samples. CirComPara2 was run using the default settings vs. all of its individual constituent methods, outperforming each method (0.86 median sensitivity, 0.91 median F1 score) with a negligable loss in precision compared to the best performing individual method (0.01 median reduction). In summary, the work by Gaffo et al. 2022 highlights the utility of a combinatorics approach to circRNA identification, mitigating the challenge of false negatives and false positives encountered by researchers.

##### Vromman et al. 2023

The authors invited the developers of 16 circRNA identification tools (CIRCexplorer3 [86], CirComPara2 [92], circRNA_finder [27], circseq_cup [98], CircSplice [99], circtools [97], CIRI2 [101], CIRIquant [106], ecircscreen (unpublished), find_circ [22], KNIFE [119], NCLScan [123], NCLcomparator [122], PFv2 [131], Sailfish-cir [140] and Segemehl [134]) to detect circRNAs using their own tool given a dataset of three deeply sequenced total RNA cancer cell lines. Of particular note in this work is the evaluation of each circRNA identification tools using an orthogonal approaches: quantitative reverse transcription polymerase chain reaction (RT-qPCR), RNase R treatment and amplicon sequencing. In agreement with previous works, the authors found that the number of detected circRNAs varies between tools, the majority of circRNAs (86%) are characterised by low BSJ counts ($\le$5) and each tool predicts differing sets of circRNAs.

For external validation, the authors aimed to select 80 highly expressed circRNAs (BSJ $\ge$5) and 20 lowly expressed circRNAs (<5) for each tool. After removing duplicate circRNAs selected by chance, the authors arrived at a final set of 1516 circRNAs. Using RT-qPCR, 1479 (97.6%) could be validated. Low abundance circRNAs exhibited higher variance in individual tool precision (median 95%, range 80-100%) compared to high abundance circRNAs (median 98.8%, range 90-100%). With respect to RNase R^+^ treatment, 112 circRNAs were discarded due to low abundance in the RNase R^-^ samples. Of the remaining 1404 circRNAs, 1319 (93.9%) were validated using RT-qPCR on RNase R^+^ samples. Highly abundant circRNAs had high RNase R^+^ precision (median 96.3%, range 74-100%) whilst lowly expressed circRNAs had lower precision (median 86.7%, range 50-100%). Generally, the precision rates calculated using RNase R^+^ is similarly high amongst tools. Amplicon sequencing was performed on a random subset (1179) of the 1516 circRNAs, with 86% readily validated using this technique. Echoing previous results, highly expressed circRNAs were more readily validated (median 95.5%, range 30-100%) compared to lowly expressed circRNAs (median 73.3%, range 17-94%).

The authors next postulated that external validation techniques be used in concert, evaluating to what extent each method supports the other. Of the 1103 circRNAs available for all three methods, 957 passed all validations, 128 failed one of two validation methods, whilst 18 failed all three. These results were used to generate the compound precision for each tool whereby true positives represent circRNAs validated by three methods and false positives represent circRNAs that failed any one validation method. The theoretical number of true positives was then calculated by multiplying the compound precision by the number of circRNAs detected by a tool. By using a high quality set of circRNAs, the authors could discern what computational strategies produce the most reliable results.

Interestingly, circRNAs containing canonical splice site signals surrounding the BSJ site and circRNAs originating from regions with an annotated linear RNA have a higher chance of being successfully validated. Furthermore, pseudo-based reference approaches exhibited higher validation rates over segmented-based approaches, whilst single-exon circRNAs had lower validation rates than multi-exon circRNAs. Additonally, tools that report circRNAs surrounded by canonical splice sites showed higher sensitivity. In terms of combinatorial approaches to circRNA identification, Vromman et al. revealed circRNAs detected by at least two tools (the default setting for CirComPara2 and nf-core circrna) had a higher chance of being validated. However, this method is not without flaws, as 0.5% failed all three validation methods and 9.9% failed at least one method. Finally, the authors investigated different combinations (pairs and triples) of tools. The findings were highly pertinent to users considering an integrated analysis approach: 1) Combining a pseudo-reference based tool with a segmented-based tool yielded a 61.1% median increase in the number of detected circRNAs vs. 35.4% when using two tools with the same alignment strategy and 2) Combining tools with differing splice site settings (canonical vs. non-canonical) yields a 76.2% median increase in the number of detected circRNAs vs. 32.6% when using tools with the same splice site settings.

The works described above point to the most common challenges facing researchers identifying circRNAs in silico. The high variance in sets of circRNAs called by individual tools, the necessity of employing multiple tools to increase sensitivity and the utility of combinatorial approaches to circRNA identification are key points to consider when designing/choosing a circRNA identification pipeline.

## Differential expression analysis

Once the circRNA transcriptome has been characterised in samples, it is often the goal of researchers to perform differential expression analysis (DEA) between phenotypes of interest using the generated circRNA count matrix. DEA can be performed manually using popular tools such as DESeq2 [176], EdgeR [177] and limma-voom [178]. Both DESeq2 and EdgeR fit a negative binomial distribution to the counts matrix and use generalized linear models to perform statistical tests, whereas limma-voom computes observational weights for a linear model using mean-variance relationship between samples on the logarithmic scale. A common filtering step prior to DEA is to require $\ge$2 reads spanning the BSJ site of quantified circRNAs. Whilst this will result in a count matrix with higher confidence circRNAs, there remains the problem of multiple zero values present in columns (samples) in which the high confidence circRNAs were not detected resulting in a sparse matrix. In our experience, providing a sparse matrix to the DESEq2/EdgeR/limma-voom packages will result in an error when calculating the library size factors for normalization. To remedy this, we suggest applying a pseudocount to the sparse matrix prior to performing DEA.

A major factor of DEA that has only recently been considered is the increasingly popular use of multiple quantification tools to generate the final count matrix [92, 124, 147]. This comes with the upside of increasing the recall rate of the quantification analysis by overlapping the calls of multiple quantification tools, however the number of called reads spanning the BSJ site for a circRNA are likely divergent across the quantification tools employed [146, 147]. This presents the issue of which function to apply when consolidating reads from multiple tools; should researchers average circRNA expression across multiple tools? Perhaps they may be inclined to take the maximum read count value returned for a circRNA. Regardless of the function applied, there will at the very least be a loss of information and at worst, a significant overestimation of a circRNAs expression by selecting highly expressed outliers. To overcome this issue, Buratin et al. (2022) [179] perform DEA by modelling the effect of the phenotype of interest whilst simultaneously modelling the variance of circRNA reads between different quantification tools as a random effect using generalized linear mixed models e.g: $\sim$ phenotype group + (1$\vert$quantification tool 1) + (1$\vert$quantification tool 2) etc. In this manner, one can obtain robust differentially expressed circRNAs estimates without discarding any of the information obtained from mutliple quantification tools. We recommend users adopt this approach when using a consensus based approach to calling circRNAs, a method that has been shown to increase accuracy in the quantification step [147].

Considerable efforts have been made to automate the process of differential expression analysis of circRNAs for researchers. CIRIquant and nf-core circrna ([106, 124]) offer automated differential expression analysis of circRNAs using edgeR and DESeq2, respectively. The main drawback of using automated differential expression analysis pipelines are the constraints placed on the complexity of the model design; these methods are only capable of analyzing the predictor variable whilst controlling for the effect of covariates, and do not facilitate more complex designs with additive, interactive or nested effects. For complex designs, we recommend users perform differential expression analysis manually.

Finally, CIRI, CIRI2, CIRIquant, Sailfish-cir and DCC (via the CircTest module) are all capable of calculating the circular RNA/linear RNA ratio at a junction site [73, 100, 101, 106]. This ratio can be used to perform differential splicing analysis between samples of interest to identify conditions in which transcripts favour circularization over canonical linear splicing. CIRIquant and CircTest automate this process for users, greatly reducing the time required to perform the analysis. CIRIquant can directly perform differential splicing analysis between two samples using the ‘CIRI_DE’ module via the rate-ratio.test R package [180]. CircTest offers the distinct advantage over CIRIquant of being able to perform differential splicing analysis between multiple samples. Users can apply the CircTest module directly to the output directory of DCC, or manually supply previously generated circRNA count matrices and linear RNA count matrices in addition to a phenotype file with sample descriptions. CircTest uses a beta binomial distribution to model the data (circRNA/circRNA + linear RNA) and performs an ANOVA test to identify differential splicing events between conditions using the AOD R package [181]. We stress that users only perform differential splicing analysis using total RNA-Seq datasets, as RNase R libraries deplete linear RNA.

## circRNA interactions

### ceRNA networks

**Fig. 3:**
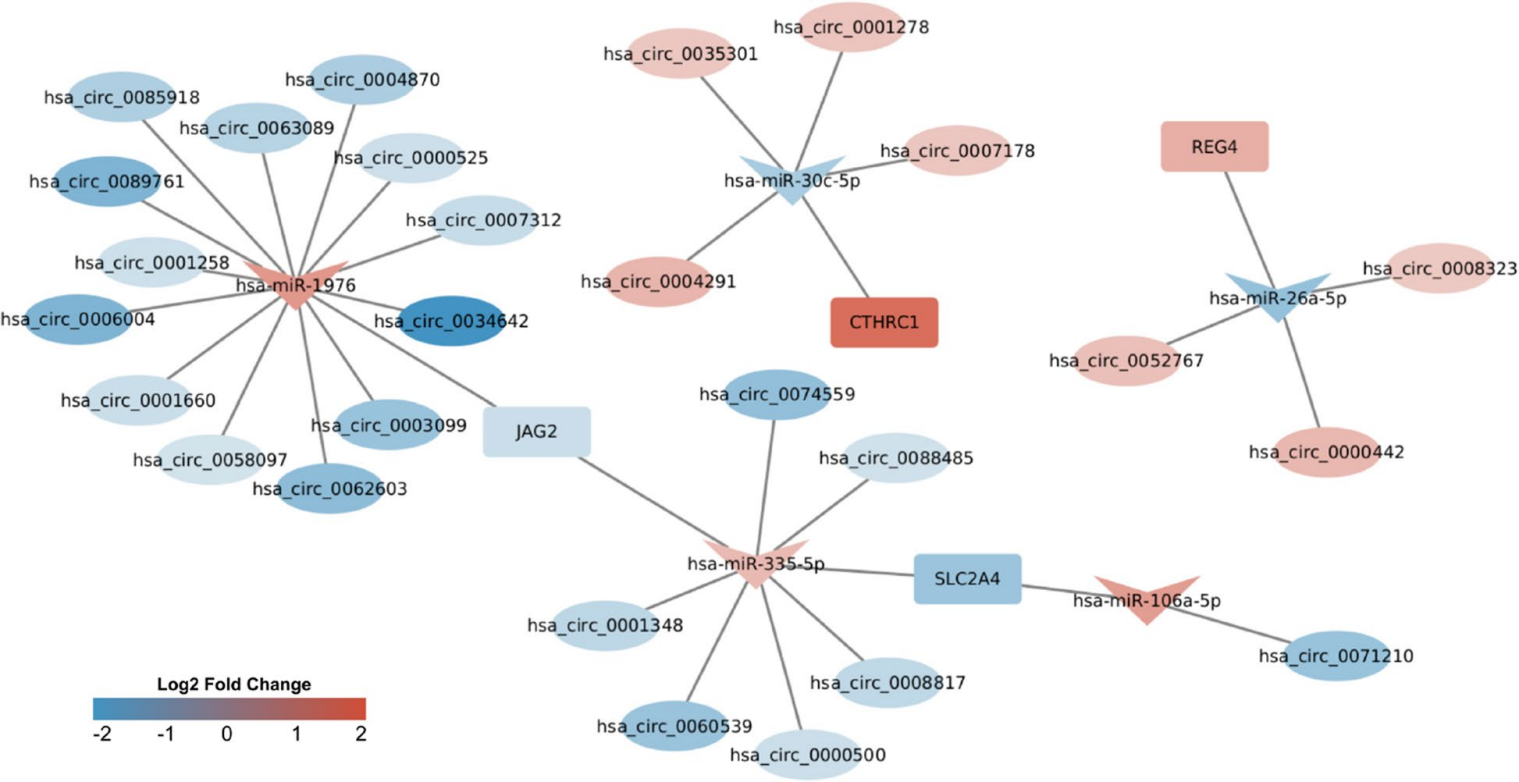
Cytoscape visualisation of a ceRNA network. CircRNAs are represented as ellipse nodes, miRNAs as arrow nodes and mRNAs as rectangular nodes. Edges represent interactions predicted by both miRanda and TargetScan. The network has been filtered to select circRNA-miRNA-mRNA subgraphs representing circRNA sponging of miRNAs whereby upregulation of one biotype influences the expression of downstream targets

circRNAs can function as miRNA sponges when they enter the cytoplasm [21, 22], affecting the ceRNA network by competitively binding miRNAs and sequestering the degradation of its mRNA target. The predicted interactions of circRNA-miRNAs and miRNA-mRNAs targets can be used to create a tri-partite ceRNA network representing the circRNA-miRNA-mRNA interplay in cells (Fig. 3). Researchers can achieve this by using existing databases, performing manual predictions using sequence alignment tools against databases, or a combination of both. Several publicly available databases exist which contain predicted circRNA-miRNA interactions in downloadable files such as circBase [166] and CSCD [182]. Additionally, starBase [183] offers an API function to submit requests for predicted circRNA-miRNA targets. Once the circRNA-miRNA pairs have been generated, the miRNAs can be used as inputs for deriving miRNA-mRNA interactions. Given that miRNAs have been studied since the early 1990s (compared to the more recent revivial of interest in circRNAs in 2012), there exist multiple databases for predicting miRNA-mRNA pairs. miRBase [184], miRTaRBase [185], miRNet [186] and TargetScan [187] represent a selection of the available databases for this task.

To predict circRNA-miRNA and miRNA-mRNA targets manually, users can avail of multiple sequence alignment tools miRanda [188] and TargetScan [187]. The full mature spliced sequence of each circRNA can be scanned for miRNA response element (MRE) sites by passing the sequence in FASTA format to each tool. TargetScan offers the advantage of reporting each miRNA match as a 6-mer, 7-mer or 8-mer, detailing the number of matching nucleotides in the circRNA MRE site and the miRNA seed region. To reduce the number of false positives in the analysis, users can adopt three strategies: 1) remove 6-mers sites that are considered poorly conserved in comparison to 7-mer and 8-mers; 2) overlap results between miRanda and TargetScan; or 3) overlap predicted MRE sites with AGO2 binding sites. These filtering steps can be applied to circRNA-miRNA and miRNA-mRNA predictions alike. Finally, in the event expression data between phenotypes is available for circRNAs, miRNAs and mRNAs, users may wish to apply filtering to conform to the ceRNA hypothesis by selecting circRNA-miRNA-mRNAs subgraphs in which the circRNA exhibits up-regulation, its target miRNA is down-regulated and the target mRNA of the down-regulated miRNA is up-regulated. The inverse filtering expression can be applied to generate a ceRNA network modelling up- and down-regulated circRNAs. Tripartite networks can then be visualised and analysed using Cytoscape [189] and its numerous plugins for network analysis. The main challenge in performing manual circRNA-miRNA predictions is providing an accurate mature spliced sequence to each tool, details of which are discussed in “Full circle reconstruction” section.

### circRNA-RBP prediction

Whilst circRNA-miRNA binding is the most studied functionality of circRNAs, there is increasing evidence to suggest circRNAs interact with RBPs at multiple iterations of their life cycle. Quaking (QKI), FUS, HNRNPL, RBM20 and Muscleblind are all RBPs which bind to specific intronic motifs, promoting the formation of circRNAs [25, 44, 45, 190, 191], whilst ADAR1 and DHX9 have been shown to destabilize inverted Alu repeats, supressing back-splicing [159, 192]. *CircPABPN1* has been shown to modulate the transcription of its cognate mRNA *PABPN1* by competitively binding and reducing the availability of HuR, a translational activator protein [193]. Additionally, *circFoxo3* binds p21 and CDK2 RBPs, forming ternary complexes inhibiting cyclin E/CDK2 complex formation, arresting cells in G1 phase [194].

circRNA-RBP interactions can be characterised using cross-linking and immunoprecipitation (CLIP-seq) datasets however, the assay suffers from limitations. Firstly, CLIP-Seq reads are produced via enzymatic degredation, producing single-end reads of length <50bp. These short, single-end reads are unsuitable for traditional circRNA identification tools developed for RNA-Seq data which suffer from poor mapping estimates when using short reads and in the absence of paired-end reads, will generate high rates of false-positives. To accurately identify circRNAs in CLIP-Seq data, researchers can use Clirc, a computational tool capable of detecting BSJ sites bound to RBPs [195]. Clirc collapses reads to remove PCR duplicates prior to constructing a psuedo-reference based on publicly available human, mouse and drosophila circRNAs and circRNAs detected in ENCODE datasets using CIRI2. Reads that contiguously align to the reference genome are discarded, whilst reads mapping to the pseudo-reference are indicative of BSJ sites in circRNAs. The authors concede that Clirc is constrained to detecting circRNAs in the pseudo-reference and cannot detect circRNAs *de novo*. Additionally, Clirc can only detect RBPs that directly bind to the BSJ site as distinguishing RBPs binding to ‘linear’ sequences in circRNAs/mRNAs remains intractable.

Databases such as CircInteractome [196] and starBase [183] host results of circRNA-RBP interactions validated using CLIP-Seq experimental data. Due to the costs associated with CLIP-seq, there have been several computational methods developed to predict circRNA-RBP interactions by analysing motif sequences. CircRNAs Interact with Proteins (CRIP) is tool that represents circRNA-RBP interactions as a binary classification problem. The authors have developed a novel sequence encoding scheme whereby RNA triplets are represented as pseudo-amino acids, one-hot encoded and passed to a convolutional neural network (CNN) and a bidirectional long- and short-term memory (LSTM) network to exploit sequence information of 37 RBPs and the corresponding 32,216 circRNAs they bind [109]. Source code and training data are freely available, allowing users to leverage the information provided by CircInteractome to predict circRNA-RBP interactions using their own circRNA sequence data. This does however, require a high degree of computational competency to run, in which case users may find CirRBP [107] a more suitable alternative. CirRBP utilizes a stacked ensemble deep learning model to predict RBP binding sites within a user supplied circRNA sequence, sourcing circRNA-RBP binding information from CircInteractome, starBase and CSCD2. The authors have packaged the underlying algorithm and models used for CirRBP as a publicly available webserver [197] greatly reducing the computational barrier to entry for researchers to perform circRNA-RBP predictions.

## circRNA databases

Multiple circRNA databases currently exist providing users with circRNA annotations, predicted interactions, mature spliced sequence and expression estimates across cell lines (Table 2). Typically, these databases are derived from a selection of published ribosomal depleted RNA-Seq datasets [19, 20, 22, 25, 29, 76, 159, 198] and are processed using a circRNA identification pipeline. It is worth noting that there is no universal ‘gold standard’ pipeline for circRNA identification, thus each database will vary in their results. For example, circBase [166] and CIRCpedia exclusively use find_circ and CIRCexplorer2, respectively, whilst CSCD2 [199] employs CIRI2, CIRCexplorer2, circRNA_finder and find_circ to produce its database, allowing users to identify which circRNAs have been called by multiple tools. Other databases such as circRNADb [165] host circRNA annotations collated from published literature, removing biases inherent to specific pipelines. With respect to the fucntional interactions of circRNAs, the starBase [183] and TRCirc [200] databases contain RNA-RNA interactions and RNA-protein interactions using CLIP-Seq and CHIP-Seq data, respectively. Researchers can also search disease specific circRNAs backed by experimental findings in published literature via Circ2Disease [201].

**Table 2:** Online databases for circRNAs

Database	Data available	Organisms^a^	Reference
AtCircDB	A. thaliana circRNAs, miRNA targets	ath	[202]
circAtlas	circRNA sequences, conservation score, miRNA & RBP targets, isoforms, expression in tissues, junction ratio, reported diseases	hsa, mml, mmu, rno, ocu, clf, fca, ssc, oar, gga	[169]
circBase	circRNA sequences, circRNA expression in cell lines/tissues	hsa, mmu, cel, lch, lme	[166]
CircBank^b^	miRNA targets, m^6^A modifications, conservation, mutations and coding potential	hg19	[203]
CircInteractome	miRNA & RBP targets, primer design, siRNA sites	hsa	[196]
CircNet	miRNA & RBP targets, ceRNA networks construction, coding potential	hsa	[204]
CircFunBase	circRNA predicted function, miRNA & RBP targets, visual representation of ceRNA network	ath, osa, tae, sly, gsp, hvu, ade, hsa, mml, rno, mmu, gga, ssc, bta, dme, ocu	[205]
CIRCpedia	circRNA exprression in cells and tissues	hsa, mmu, cel, dme, dre, rno	[206]
CSCD2	circRNAs in cancer, target miRNAs & RBP, coding potential	hsa	[199]
circRNADb	exonic circRNAs, coding potential	hsa	[165]
CircRiC^b^	circRNAs in cancer	hsa	[207]
Circ2Disease	circRNAs associated with diseases	hsa	[201]
CircR2Disease	circRNAs associated with diseases	hsa	[208]
Circ2Traits^b^	circRNA-miRNA disease associations	hsa	[209]
circRNADisease^b^	circRNAs associated with diseases	hsa	[210]
CropCircDB	circRNAs in maize & rice	osa, zma	[211]
DeepBase	circRNA, lncRNA, miRNAs in tissues and cancers	hsa	[212]
exoRBase	Atlas of mRNAs, lncRNAs,& circRNAs in extracellular vesicles	hsa	[213]
MiOncoCirc	Compendium of circRNA datasets in cancer	hsa	[214]
NeuroCirc	circRNA expression in brain regions, circQTLs	hsa	[215]
PlantCircNet^b^	ceRNA regulatory networks	ath, gma, hvu, osa, sly, tae, zma, bdi	[216]
PlantcircBase	circRNAs in plants, ceRNA regulatory network	ath, gma, hvu, osa, sly, tae, zma, gar, ghi, gra, ptr, stu, csi, nbe, pbe, osi	[217]
starBase	RNA-RNA interactions based on CLIP-Seq data	23 species	[183]
TRCirc	Transcriptional regulation of circRNAs using CHIP-Seq data	hsa	[200]

^a^Species abbreviations: ade, *Actinidia deliciosa*; ath, *Arabidopsis thaliana*; bdi, *Brachypodium distachyon*; bta, *Bos taurus*; cel, *Caenorhabditis elegans*; csi, *Camellia sinensis*; dme, *Drosophila melanogaster*; dre, *Danio rerio*; gar, *Gossypium arboreum*; gga, *Gallus gallus*; ghi, *Gossypium hirsutum*; gma, *Glycine max*; gra, *Gossypium raimondii*; gsp, *Gossypium spp.*; hsa, *Homo sapiens*; hvu, *Hordeum vulgare*; lch, *Latimeria chalumnae*; lme, *Latimeria menadoensis*; mml, *Macaca mulatta*; mmu, *Mus musculus*; nbe, *Nicotiana benthamiana*; ocu, *Oryctolagus cuniculus*; osa, *Oryza sativa*; osi, *Oryza sativa ssp. indica*; pbe, *Pyrus betulifolia*; ptr, *Poncirus trifoliata*; rno, *Rattus norvegicus*; sly, *Solanum lycopersicum*; ssc, *Sus scrofa*; stu, *Solanum tuberosum*; sly, *Solanum lycopersicum*; tae, *Triticum aestivum*; zma, *Zea mays*

^b^URL not accessible at time of drafting review

One of the key challenges facing researchers when using circRNA databases is the lack of a standardised naming format for circRNAs. Chen et al. (2023) [218] use the example of the functional *FAM20A* circRNA to depict the wide discrepancies between nomenclature: HSA_CIRCpedia_64725 in CIRCpedia, hsa-FAM120A_0006 in circAtlas, hsa_circFAM120A_007 in circBank, and hsa_circ_0001875 in circBase. With ‘hsa’ being the only common string between the four identifiers, Chen et al. proposed a novel naming convention for circRNAs. They provide examples for classic exonic circRNAs, EIcircRNAs and ciRNAs: 1) *circCOX5A(2,3)* an exonic circRNA derived from the *COX5A* gene that uses exons 2 and 3; 2) *circCAMSAP1(2,RI,3)* an exonic-intronic circRNA derived from the *CAMSAP1* gene that uses exons 2 and 3, with a retained intron (RI) between exons 2 and 3; 3) *ciANKRD52(2)* an intronic circRNA derived from the *ANKRD52* gene that retains the second intron. CircAtlas (version 3.0) [219] provides users with mapping keys between circRNA positional, circBank, circBase and CIRCpedia identifiers to the latest ’uniform ID’ values suggested by Chen et al. (2023). Whilst we agree that the standardisation of circRNA identifiers is a useful endeavour, we believe it may take several years to adopt. The main hurdle we observe is that the most popular circRNA identification tools that are currently in use by researchers are solely based on identifying BSJ reads in RNA-Seq data. These tools cannot fully resolve the internal structure of circRNAs, thus researchers are not able to accurately annotate internal exon/intron usage required to conform to the proposed naming convention. Secondly, circBase, CSCD2 and CIRCpedia remain hugely popular databases for circRNAs. Finally, the research community must agree on the reference used for reporting circRNAs (e.g ENSEMBL [220], RefSeq [221]) and make clear which reference annotation files were used for circRNA annotation in order to make results reproducible.

## Concluding remarks

circRNAs are a class of non-coding RNAs which are gaining recognition for their roles in cellular processes as transcriptional regulators. Despite circRNAs representing an increasingly popular area of research, there still remain several challenges in accurately characterising circRNAs computationally. This is perhaps most apparent in a subset of widely utilised circRNA identification tools that are entirely predicated on detecting the BSJ of circRNAs in sequencing data. Whilst these tools are useful for detecting and quantifying circRNAs, they struggle to fully resolve the full circRNA sequence or delineate circRNA isoforms generated from the same BSJ. Furthermore, these tools suffer from varying rates of sensitivity and depending on the underlying methods used, can be prone to false positives. Integrated methods have been developed to address this issue however, these tools can hardly be considered a panacea given recent studies have shown circRNAs called by multiple tools can fail external validation. The development of a gold standard set of circRNAs is a necessary step to benchmark the performance of current and future circRNA identification tools, diagnosing their inherent blind spots. Another striking absence in the field of circRNA research is the lack of a reference GTF/GFF file of known circRNAs. In our opinion this goes hand in hand with the development of a gold standard set of circRNAs and the unification of circRNA nomenclature. Once developed, the research community can report circRNAs in a consitent manner and develop rapid pseudo-alignment based tools mimicking those in the space of RNA-Seq (Kallisto [222], Salmon [223]). Third-generation sequencing represents a promising avenue for full circle characterisation of circRNAs and accurate prediction of interactions with miRNAs and RBPs. However, few computational tools consider the final tertiary structure of circRNAs which can greatly influence its capacity to bind miRNAs and RBPs or form scaffold structures.

In conclusion, our work provides an accessible guide for researchers to navigate the landscape of computational circRNA research. We have provided a comprehensive overview of the tools available for circRNA identification, full circle reconstruction, differential expression analysis, circRNA interactions and databases, highlighting the limitations of current tools and suggesting solutions to common pitfalls.

## Data Availability

No datasets were generated or analysed during the current study.
